# Implementation of the In Vitro Seizure Liability Assay (iLA^seizure^ ) in Drug Discovery and Development: Mechanism of Action Case Studies

**DOI:** 10.1002/prp2.70317

**Published:** 2026-09-08

**Authors:** Kimberly Rockley, Ruth Roberts, Hannah Jennings, Karen Jones, Magali‐Anne Maizières, Michael Morton

**Affiliations:** ^1^ ApconiX Macclesfield UK; ^2^ Department of Biosciences University of Birmingham Edgbaston UK

**Keywords:** central nervous system, drug development, drug discovery, ion channels, neurons, seizures, stem cells

## Abstract

Central nervous system (CNS) liabilities remain a significant cause of attrition during small molecule drug development. We previously developed the in vitro seizure liability assay (iLA^seizure^), a new approach methodology (NAM) comprising a human induced pluripotent stem cell neuron microelectrode array (MEA) assay coupled with a panel of human ion channels associated with CNS perturbation and especially seizure, evaluated by electrophysiology. Here, we present a series of illustrative case studies demonstrating the potential applications of iLA^seizure^. Amoxapine (shows clinical seizures) gave concentration‐dependent changes in MEA parameters and inhibited seizure‐related ion channels with high potency (IC_50_ < 30 μM) at the human therapeutic concentration range (0.57–1.91 μM). Comparison of seizure risk in five compounds in a lead series showed clear ranking, with no changes in any of the MEA parameters for one of the five compounds. In a second case study, there were dog‐specific seizures in good laboratory practice (GLP) toxicology: this was explained by a clear seizurogenic phenotype at 100 μM in MEA for a dog‐specific metabolite and a hit on the ion channel panel at NMDA1/2A with high potency (IC_50_ of 19.3 μM). In the third case study, one clinical candidate showed no changes in MEA parameters at the clinical C_max_ of 1 μM, whereas the other clinical candidate showed a potent seizurogenic phenotype with changes in many MEA parameters at 3–10 μM, correlating with the clinical C_max_ of 5 μM. Overall, iLA^seizure^ offers new opportunities to predict, understand, and avoid CNS side effects such as seizures in drug discovery and development, before animals, resources, and time have been wasted. This work should be considered within the context of recent proposed updates to safety pharmacology guidelines, such as ICH S7A. However, further work is needed to validate assay performance and clarify the context of use.

**Abbreviations**
CDER: center for drug evaluation and research
CNS: central nervous system
COU: context of use
FDA: food and drug administration
GLP: good laboratory practice
hiPSC: human induced pluripotent stem cells
iLA: integrated In Vitro CNS liability assay
MEA: microelectrode array
NAM: new approach methodology
NMDA1/2A: N‐methyl‐D‐aspartate receptor 1/2A

## 1 Introduction

Central nervous system (CNS) toxicity, and specifically drug‐induced seizures, are a frequent occurrence during small molecule drug discovery and development [1, 2], leading to project delays and failures. Compounds associated with seizure liability span a wide variety of therapy areas, including CNS, cardiovascular, gastrointestinal, respiratory, and inflammation [1, 3, 4]. Seizures are usually detected during preclinical rodent and non‐rodent studies or even later, once the drug has entered clinical development, leading to a significant waste of time and resources. Some progress has been made in seizure detection using automated video systems that record and analyze animal movements [5], and a lot of effort has been invested in developing screening methods that could be used earlier in drug discovery, such as the hippocampal brain slice [6] or zebrafish larval locomotor assays [7]. However, these methods have had limited success, with concerns regarding throughput and translation of rodent and zebrafish data to humans.

The in vitro liability assay (iLA) is based on measuring electrical activity in induced human pluripotent stem cell (hiPSC) neurons using a microelectrode array (MEA), coupled with a neuronal ion channel panel [8]. iLA was developed as an in vitro CNS and seizure screening assay for potential new drugs. Activity in MEA is based on evaluating electrical spikes and bursts measured in individual cells and in the overall cellular network. Activity at ion channels is based on evaluating an IC_50_ for inhibition as described previously [8], then allocating a “potency category” as has been done for hERG [9, 10]. Here, we report the outcome of using this framework to quantify seizure‐like parameters for a known seizurogenic compound (amoxapine) followed by the evaluation of several unknown compounds to determine the utility of iLA both in optimal drug design and in problem solving. Emphasis was placed on in vitro to in vivo translation of exposures and on mechanistic information gained from ion channel inhibition.

## 2 Materials and Methods

MEA was conducted on hiPSC neurons (Fujifilm Cellular Dynamics Inc.) using the Axion Maestro MEA instrument as described previously [8]. Briefly, glutaneurons (GNC) containing a ratio of approximately 80% glutamatergic neurons and 20% GABAergic neurons were mixed with astrocytes (ASC) to create a final culture containing 68% glutamatergic neurons, 17% GABAergic neurons and 15% astrocytes. This cell ratio was kept as consistent as possible to minimize variability. The cells were maintained for 21–25 days in culture to form functional networks before testing compounds. Before compound addition, the MEA plate was equilibrated in the MEA Pro instrument (Axion Biosystems) for at least 15 min. Then, 15‐min baseline recordings were taken. After a 1‐h incubation with test compounds, 15‐min treatment recordings were taken and loaded onto the Axion Neural Metric Tool for analysis of the spikes and generation of raster plots. Raster plots were quantified according to the parameters listed in Table 1. Stock compounds were solubilized at 1000× their top concentration in DMSO. Serial dilutions were prepared at 10× final concentration, which were added directly to media in the wells. A 0.1% DMSO vehicle control was included on every plate. Spike files were created from the original 15‐min recordings for analysis. Raster plots of the first 150 s of the recordings were created using the Axion Neural Metric Tool (v4.1.1), and the effects of compound treatment on selected MEA parameters were determined using the Axion Axis Metric Plotting Tool (v2.5.1). The effect of each drug was measured relative to its own pre‐drug baseline (from the same well). Compounds or vehicle were applied directly to wells, and the average percentage change from baseline was calculated. The DMSO vehicle control is presented for each parameter and usually shows minimal changes (±20%). MEA data were categorized into thresholds of no change (NC; 0%–29%), moderate change (*↑↑/↓↓;* 30%–49%) and high change (*↑↑↑/↓↓↓*; > 50%). This categorization was devised based on a combination of assay variability (typically see ±20% variability with vehicle control) and historical datasets using known seizurogenic compounds with various mechanisms of action.

**TABLE 1:** Selected MEA parameters and their definitions.

**Firing rate parameters**	
Mean firing rate (Hz)	The mean firing rate based on only electrodes with activity greater than minimum spike rate.
ISI coefficient of variation	The coefficient of variation (standard deviation/mean) of the inter‐spike interval, the time between spikes. This is a measure of spike regularity.
**Burst rate parameters**	
Burst frequency (Hz)	The total number of single‐electrode bursts divided by the recording time.
Burst duration (s)	Average time from first spike to last spike in a single‐electrode burst.
Number of spikes per burst	Average number of spikes in a single burst.
Mean ISI within burst (s)	Average inter‐spike interval, time between spikes, for spikes in a single‐electrode burst. This is a measure of burst intensity; smaller values mean more intense bursts.
**Network burst parameters**	
Network burst frequency (Hz)	Total number of network bursts divided by the recording time.
Network burst duration (s)	Average time from first spike to last spike in a network burst.
Number of spikes per network burst	Average number of spikes in a network burst.
Mean ISI within network burst (s)	Average inter‐spike interval, time between spikes, for spikes in a network burst. This is a measure of burst intensity; smaller values mean more intense bursts.

Only active electrodes (mean spike rate [MSR]_5 spikes/min) in active wells (minimum of eight active electrodes) were included in the analysis. Single‐electrode bursts were detected using an ISI threshold with a minimum of five spikes and a maximum ISI of 100 ms. Network bursts were identified using the envelope method. All MEA data constitute one biological replicate from client studies. Technical replicates (the number of individual wells) and lots of GNC and ASC used are specified within the results section.

All sodium and potassium voltage‐gated ion channel cell lines were generated internally by standard recombinant techniques. All channels were produced in a CHO‐K1 (RRID: CVCL_0214) or HEK‐293 (RRID: CVCL_0045) background at ApconiX. GABA, NMDA, and nAChR channels were purchased from B'SYS GmbH (Witterswil, Switzerland). hCaV2.1 was generated by transient transfection into HEK‐293 cells. All cell lines were free from contamination.

Automated patch clamp electrophysiology experiments (QPatch II, Sophion Biosciences, Ballerup, Denmark) were performed as previously described to determine IC_50_ values [8]. Stock compounds in DMSO were diluted to their top test concentration in HEPES‐buffered saline. Single‐cell ionic currents were measured in whole‐cell configuration at room temperature (25°C). Compounds were incubated for at least 120 s. Concentration–response curves were generated by cumulative addition of compound with concentrations low to high. In all cases, steady‐state inhibition was achieved before the next concentration of compound was added. The difference in current between the pre‐compound and post‐compound measurements was exported and analyzed using GraphPad Prism software. A minimum of 4‐point concentration–response curves were generated for each compound. IC_50_ values (half‐maximal inhibitory concentration) were obtained from a 4‐parameter logistic fit of normalized concentration–response data. Data from 3 to 6 cells were used to generate the concentration–response curves.

## 3 Results

**FIGURE 1:**
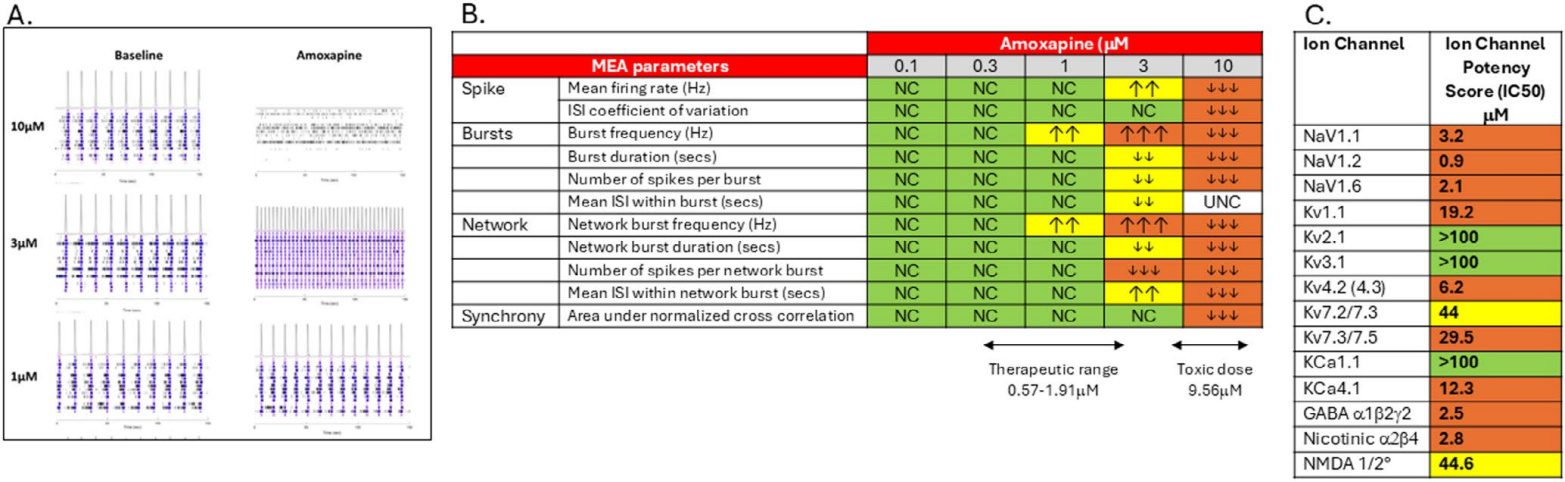
Changes in MEA parameters with Amoxapine, an antidepressant associated with convulsions. Raster plots are shown for 1, 3 and 10 μM Amoxapine compared with baseline data (C). Baseline and treatment data were compared to identify % change in selected parameters (A). UNC (unclear, possibly due to overstimulation of the cells); NC (no change) 0%–29% change; ↑↑ or ↓↓ 30%–49% change; ↑↑↑ or ↓↓↓ > 50% change. *N* = 4 technical replicates were performed for each dose of the compound. Cell lots used were GNC: 106060, ASC: 105981. For ion channel inhibition, colors depict the safety margin to the mean Amoxapine C_max_ (1 μM) of < 30 (orange), 30–100 (yellow), and > 100 (green) based on established practice for hERG [9] (B). Therapeutic and toxic plasma concentrations of Amoxapine are 0.57–1.91 μM and 9.56 μM, respectively (shown as range arrows).

Raster plots generated for the positive control amoxapine (an antidepressant with a high incidence of clinical adverse events described as seizures [11]) showed some changes at 1 μM, clear evidence of significant changes at 3 μM, and intense changes at 10 μM (Figure 1A). These visible changes in raster plots correlated with notable changes in many measured MEA parameters such as burst frequency and network burst frequency, which both showed increases beginning at 1 μM with a clear concentration–response (Figure 1A). These changes occurred at the human therapeutic concentration range (0.57–1.91 μM), providing evidence of a correlation between in vitro responses and clinical exposures. Characteristic of CNS‐active therapies, amoxapine also inhibited several seizure‐related ion channels (Figure 1B) with high (NaV1.1, NaV1.2, NaV1.6, Kv1.1, Kv4.2, Kv7.3, KCa4.1, GABA α1β2γ2, Nicotinic α2β4) or medium (Kv7.2, NMDA1/1) potency.

In the first case study of unknown compounds, it was important to evaluate seizure risk in a lead series during drug discovery lead optimization. The five lead series were evaluated by MEA (Table 2). Compound E showed marked changes in MEA parameters such as network burst frequency and the mean inter‐spike interval. Compounds B, C, and D also showed changes in network burst frequency, and compounds B and D showed changes in mean ISI. However, compound A had no changes in any of the MEA parameters and was deemed the safest lead to progress in this project.

**TABLE 2:** Changes in MEA parameters with five different lead series, A–E, all tested at 30 μM. MEA work was conducted and presented as described in Table 1. NC (no change) 0%–29%; ↑↑ or ↓↓ 30%–49% change; ↑↑↑ or ↓↓↓ > 50% change.

		Lead series being tested (all at 30 μM)				
MEA parameters		A	B	C	D	E
Spike	Mean firing rate (Hz)	NC	NC	NC	NC	NC
	ISI coefficient of variation	NC	NC	NC	NC	NC
Bursts	Burst frequency (Hz)	NC	NC	NC	NC	NC
	Burst duration (s)	NC	NC	NC	NC	NC
	Number of spikes per burst	NC	NC	NC	NC	NC
	Mean ISI within burst (s)	NC	NC	NC	NC	NC
Network	Network burst frequency (Hz)	NC	↑↑	↑↑↑	↑↑	↑↑↑
	Network burst duration (s)	NC	NC	NC	NC	NC
	Number of spikes per network burst	NC	NC	NC	NC	↓↓
	Mean ISI within network burst (s)	NC	↓↓	NC	↑↑	↑↑↑
Synchrony	Area under normalized cross correlation	NC	NC	NC	NC	NC

**FIGURE 2:**
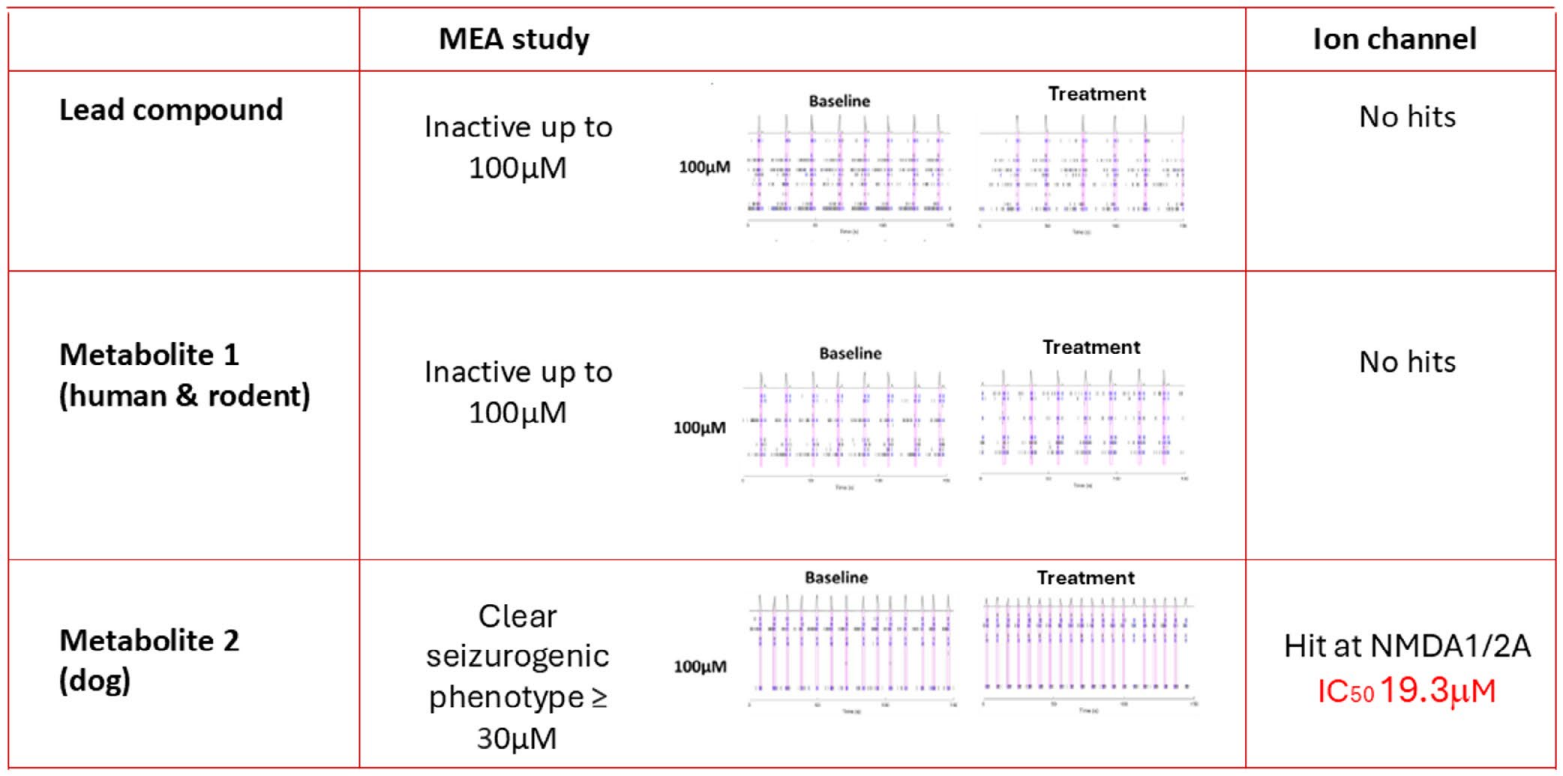
Baseline and treatment raster plots for parent drug and prevalent human/rodent or dog metabolites, alongside an ion channel panel summary. MEA and ion channel work was conducted and data presented as described in Table 1 and Table 3. For the dog metabolite, there was a hit on the ion channel panel with inhibition at NMDA 1/2A at 19.3 μM.

In the second case study, a project had been brought to a standstill by convulsions seen in dogs during the preclinical good laboratory practice (GLP) studies required to support first‐in‐human clinical trials. The question to be addressed was, “Are these convulsions relevant to humans?” Notably, there were no seizures in the rat GLP studies conducted at the same time. An evaluation of the project revealed a species difference in metabolism with one main metabolite common to humans/rats, and a different metabolite that was minor in humans/rats but the major (only) metabolite in dogs. The parent compound, the human/rat specific and the dog‐specific metabolite were evaluated in both MEA and the ion channel panel (Table 3 and Figure 2).

**TABLE 3:** Comparison of MEA parameters for A. parent drug and prevalent human/rodent (B) or dog (C) metabolites. MEA work was conducted and presented as described in Table 1. NC (no change) 0%–29%; ↑↑ or ↓↓ 30%–49% change; ↑↑↑ or ↓↓↓ > 50% change.

A.		Parent drug (μM) *				
MEA parameters		1	3	10	30	100
Spike	Mean firing rate (Hz)	NC	NC	NC	NC	NC
	ISI coefficient of variation	NC	NC	NC	NC	NC
Bursts	Burst frequency (Hz)	NC	NC	NC	NC	NC
	Burst duration (s)	NC	NC	NC	NC	NC
	Number of spikes per burst	NC	NC	↑↑	NC	NC
	Mean ISI within burst (s)	NC	NC	NC	NC	NC
Network	Network burst frequency (Hz)	NC	NC	NC	NC	NC
	Network burst duration (s)	NC	NC	NC	NC	NC
	Number of spikes per network burst	NC	NC	NC	NC	NC
	Mean ISI within network burst (s)	NC	NC	NC	NC	NC
Synchrony	Area under normalized cross correlation	NC	NC	NC	NC	NC

B.		Prevalent human and rodent metabolite (μM) *				
MEA parameters		1	3	10	30	100
Spike	Mean firing rate (Hz)	NC	NC	NC	NC	NC
	ISI coefficient of variation	NC	NC	NC	NC	NC
Bursts	Burst frequency (Hz)	NC	NC	NC	NC	NC
	Burst duration (s)	NC	NC	NC	NC	NC
	Number of spikes per burst	NC	NC	NC	NC	NC
	Mean ISI within burst (s)	NC	NC	NC	NC	NC
Network	Network burst frequency (Hz)	NC	NC	NC	NC	NC
	Network burst duration (s)	NC	NC	NC	NC	NC
	Number of spikes per network burst	NC	NC	NC	NC	NC
	Mean ISI within network burst (s)	NC	NC	NC	NC	NC
Synchrony	Area under normalized cross correlation	NC	NC	NC	NC	NC

C.		Prevalent dog metabolite (μM)				
MEA parameters		1	3	10	30	100
Spike	Mean firing rate (Hz)	NC	NC	NC	NC	NC
	ISI coefficient of variation	NC	NC	NC	NC	NC
Bursts	Burst frequency (Hz)	NC	NC	NC	NC	↑↑
	Burst duration (s)	NC	NC	NC	NC	NC
	Number of spikes per burst	NC	NC	NC	NC	↓↓
	Mean ISI within burst (s)	NC	NC	NC	NC	NC
Network	Network burst frequency (Hz)	NC	NC	NC	NC	↑↑
	Network burst duration (s)	NC	NC	NC	NC	NC
	Number of spikes per network burst	NC	NC	NC	NC	↓↓
	Mean ISI within network burst (s)	NC	NC	NC	NC	↑↑↑
Synchrony	Area under normalized cross correlation	NC	NC	NC	NC	↓↓

* No hits on ion channel panel.

The dog‐specific metabolite had a clear seizurogenic phenotype at 100 μM with changes in many MEA parameters such as burst frequency, number of spikes per burst, and most notably, mean interspike interval. In contrast, there were no changes in MEA parameters for the parent compound and the human/rat metabolite, with the exception of a small increase in the number of spikes per burst for the parent drug at 10 μM, with no concentration–response relationship. When the three compounds were examined in the ion channel panel (Figure 2), the dog‐specific metabolite gave a hit on the ion channel panel at NMDA1/2A with high potency (IC_50_ of 19.3 μM), whereas there were no hits with the parent compound or the human/rat metabolite.

In the third case study, the aim was to compare two clinical candidates for seizure risk to guide prioritization and decision‐making (Table 4). The two candidates X and Y were evaluated in MEA; compound Y showed a potent seizurogenic phenotype, with changes in many MEA parameters at 3 and 10 μM. This correlated with the clinical C_max_ of 5 μM, demonstrating relevance of these MEA data to the clinic. Candidate Y resulted in complete disintegration of synchronous firing at > 30 μM, making the raster plots unreadable. In contrast, clinical candidate X showed no changes in MEA parameters at the clinical C_max_ of 1 μM, although there were some changes at higher concentrations of > 10 μM (network interburst interval (IB) and number of spikes per network burst at > 30 μM). Overall, the data demonstrated that clinical candidate X carried a lower risk of seizure, especially considering the clinical C_max_.

**TABLE 4:** Changes in MEA parameters for two different clinical candidates, *X* and *Y*. MEA work was conducted and presented as described in Table 1 and the legend to Figure 1. NC 0%–29%; ↑↑ or ↓↓ 30%–49% change; ↑↑↑ or ↓↓↓ > 50% change.

		Compound X				
MEA parameters		1 μM	3 μM	10 μM	30 μM	100 μM
Spike	Weighted mean firing rate (Hz)	NC	NC	NC	NC	NC
	Interspike Interval (ISI) Coefficient of variation—Avg	NC	NC	NC	NC	NC
Bursts	Burst frequency—Avg (Hz)	NC	NC	NC	NC	NC
	Burst duration—Avg (s)	NC	NC	NC	NC	NC
	Number of spikes per burst—Avg	NC	NC	NC	NC	NC
	Mean ISI within burst—Avg (s)	NC	NC	NC	NC	NC
Network Bursts	Network burst frequency	NC	NC	NC	NC	NC
	Network burst duration—Avg (s)	NC	NC	NC	NC	NC
	Number of spikes per network burst—Avg	NC	NC	NC	↓↓	↓↓
	Mean ISI within network burst—Avg (s)	NC	NC	NC	NC	↑↑
	Network IBI Coefficient of variation—Avg	NC	NC	↓↓	↑↑↑	↓↓↓
Synchrony	Area under normalized cross‐correlation	NC	NC	NC	NC	NC
		C_max_ 1 μM				

		Compound Y				
MEA parameters		1 μM	3 μM	10 μM	30 μM	100 μM
Spike	Weighted mean firing rate (Hz)	NC	NC	↓↓↓	X	X
	Interspike Interval (ISI) Coefficient of variation—Avg	NC	NC	NC	X	X
Bursts	Burst frequency—Avg (Hz)	↑↑	↑↑↑	NC	X	X
	Burst duration—Avg (s)	NC	NC	↓↓	X	X
	Number of spikes per burst—Avg	NC	NC	NC	X	X
	Mean ISI within burst—Avg (s)	NC	NC	NC	X	X
Network Bursts	Network burst frequency	NC	↑↑↑	NC	X	X
	Network burst duration—Avg (s)	NC	NC	↓↓↓	X	X
	Number of spikes per network burst—Avg	NC	↓↓	↓↓↓	X	X
	Mean ISI within network burst—Avg (s)	NC	NC	↑↑	X	X
	Network IBI Coefficient of variation—Avg	NC	↓↓	NC	X	X
Synchrony	Area under normalized cross‐correlation	NC	NC	NC	X	X
				C_max_ 5 μM		

In a fourth case study, the challenge was to evaluate the in vitro to in vivo translation for the MEA assay (Table 5). Compound B was chosen for this evaluation due to the availability of in vivo data, including dose administered and resulting total plasma concentrations. Compound B had no effect on MEA parameters at concentrations of up to 10 μM (correlating with no CNS signs at total plasma concentrations of up to 38 μM), but showed marked changes at 100 μM (correlating with convulsions in vivo at total plasma concentrations of 148–297 μM).

**TABLE 5:** Evidence of good in vitro to in vivo translation for Compound B. Changes in MEA parameters are shown at 1–100 μm. Blue and red arrows depict the range of concentrations where there were no clinical signs or there were convulsions in vivo, respectively. MEA work was conducted and presented as described in Table 1. NC (no change) 0%–29%; ↑↑ or ↓↓ 30%–49% change; ↑↑↑ or ↓↓↓ > 50% change.

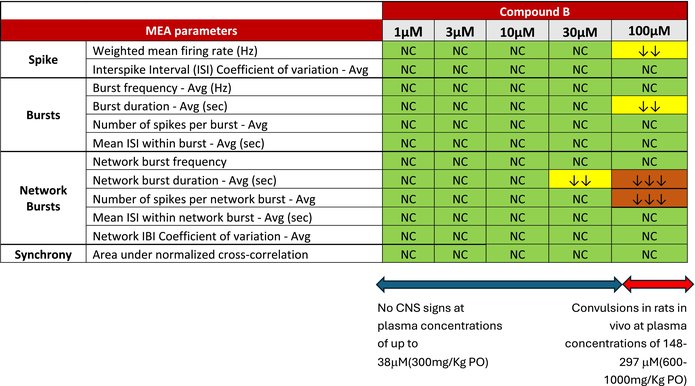

## 4 Discussion

The data presented demonstrate the potential utility of a human‐specific new approach methodology (NAM) for detecting seizurogenic potential before animals, resources and time have been wasted. Ideally, the assay should be used during lead identification and optimization to identify and deselect those compounds with seizurogenic potential. In this, the assay could be a complementary or human‐relevant alternative to the zebrafish and rat brain slice assays since these have challenges in throughput and in translation to humans.

There are several limitations of this study. The first is that it is based on client case studies and therefore data are limited and compounds are anonymous. Nonetheless, the study represents real‐life examples of challenges faced with CNS safety and potential NAM‐based solutions. Secondly, the results could reflect non‐specific CNS pharmacology or generalized network perturbation rather than being specific for seizure liability. However, non‐specific CNS pharmacology or generalized network perturbation would give a different fingerprint/pattern of activity and the benchmark validation set used in this study represents an industry standard for seizure. Thirdly, we do not yet have sufficient data to develop a specified decision framework. Exposure metrics are also uncertain. Thus, the current work is hypothesis‐generating and provides a starting point for generating context of use (COU) alongside understanding predictive human relevance and replacement value. Nonetheless, there is utility in drug discovery chemistry, comparing seizure risk for a novel drug to that of an approved drug, or characterizing the seizure risk of novel metabolites. As recently highlighted in an FDA CDER review on NAMs, assays such as iLA could be expressed as a COU statement such as, “Use of a battery of in vitro ion channel functional assays to predict in vivo human C_max_ levels of small molecule drugs associated with increased risk of seizures (or absence of seizures)” [12].

Regarding COU (see Table 6), the hiPSC‐neuronal co‐culture MEA assay is a drug development tool used to assess the ability of small molecule candidate drugs to alter human neuronal excitability. Human ion channel assays are a drug development tool used to assess perturbations to neuronal ion channel function by small molecule candidate drugs. Both assays represent biological endpoints associated with seizure risk. These assays generate data to support compound prioritization and to support the FTIH submission, providing greater assurance of CNS safety within regulatory decision‐making. These studies are part of a weight of evidence (WoE) approach to predict seizure risk when combined with other preclinical studies. Future work will develop these ideas and continue to better define appropriate COUs.

**TABLE 6:** Context of use (COU) for the MEA assay and ion channel assays in drug discovery. MEA: Microelectrode array; WOE: Weight of evidence.

Purpose of study	Timing of study	Example in paper	Notes
Prioritization: Identification of candidates without seizure liability risk ✓MEA ✓Ion channel	Lead optimisation/lead generation	Table 2	Can be used before or in place of zebrafish, primary rodent cell assays and hippocampal slice assays in early preclinical screening to identify and design out seizurogenic risk.
Problem solving: Assess relevance of convulsions observed in nonclinical and clinical studies	GLP toxicology phase	Tables 3, 4, 5	Can be used alongside data from preclinical species to problem solve and support a WoE approach
✓MEA			
Mechanistic insight: Provide mechanistic insight for convulsions observed in nonclinical and clinical studies	GLP toxicology phase	Figures 1 and 2	Can be used alongside data from preclinical species to problem solve and support a WoE approach
✓Ion channel			

With the growing focus on NAMs and the use of assays such as iLA, one key question is how in vitro findings should be interpreted in the context of human‐relevant exposures or doses. We have begun to address this point by comparing our in vitro IC_50_s with compounds where there is detailed exposure/seizure information. We found that both amoxapine and the fourth case study compound showed a good in vitro to in vivo translation. Of course, the limitation of this is that in vitro effect concentrations are compared with the data available (largely plasma therapeutic/C_max_ values) rather than unbound CNS levels which are hard to determine. In addition, the differences in methodology between MEA assays and ion channel assays (e.g., network of cells vs. single cell, different growth media requirements, different well plate material) mean there are likely differences in protein content and binding conditions. This may shift the exposure margins between the assays which may affect concentration comparisons.

In general, wherever possible, IC_50_s in the ion channel panel and concentration responses for MEA changes should be compared with the known or estimated human therapeutic dose. In practical terms, the therapeutic window for the candidate drug may not be known until at least clinical Phase II trials. However, as with other aspects of clinical translation, projects often make use of estimates of the therapeutic window based on modeling and/or other compounds in the series where there may be more information. Overall, in vitro data such as iLA are not intended to provide a stop/go, since the project would need to place such data into context with other data such as IC_50_ at the intended drug target. Nonetheless, by analogy with hERG, a potent IC_50_ would be an alert for further investigation. In contrast, iLA is highly effective in prioritization and in understanding issues already noted in the preclinical studies or in the clinic.

Compared with many other NAMs, adverse CNS events such as seizures depend on exposure of the CNS. For projects where brain penetration can be avoided, this would be the simplest route to avoid this risk. For CNS projects, brain exposure is required, shifting the focus to selectivity between intended target and off‐target effects such as ion channel activation. Overall, iLA provides an assessment of intrinsic hazard, allowing potency and ranking to occur as a strategy to reduce risk whatever the exposure profile.

As CNS‐active drugs are expected to show central effects, the key issue is contextualizing the seizure‐like signature with respect to relevant dose or exposure. It is not uncommon for seizure activity to emerge only at supratherapeutic concentrations, and often drug programs continue until retrospective analyses reveal that an active metabolite may have seizurogenic potential. The potential for seizure risk is hard to predict with current models, but we are working on some predictive structure–activity relationship (SAR) models for ion channel activity that will offer the potential to evaluate predicted metabolites *in silico* before any wet work is conducted [13].

The concepts outlined are aligned with the current scientific and regulatory momentum surrounding the modernization of ICH S7A [14]. Recent proposals for revising the guideline emphasize enhanced use of secondary pharmacology, NAMs, integrated risk assessment strategies, and WOE approaches, all of which could provide a stronger regulatory framework for prospective seizure liability assessment. The case studies presented here illustrate iLA being used for ranking, hazard identification, de‐selection and mechanistic investigations, as has been seen in the past for alternative models such as zebrafish and rat hippocampal brain slices. Further work in this area is needed to better define COU and exposure metrics.

## Author Contributions

**Kimberly Rockley:** conceptualization, data curation, formal analysis, methodology, investigation. **Ruth Roberts:** conceptualization, data curation, supervision, writing – original draft, writing – review and editing. **Hannah Jennings:** data curation, formal analysis, writing – review and editing, methodology, investigation. **Karen Jones:** conceptualization, data curation, formal analysis, visualization, writing – review and editing, investigation, methodology, resources. **Magali‐Anne Maizières:** conceptualization, data curation, formal analysis, visualization, writing – review and editing, software. **Michael Morton:** supervision, writing – review and editing, conceptualization, data curation, formal analysis, project administration.

## Funding

All work was funded by ApconiX or by the third‐party companies that provided the compounds for investigation.

## Ethics Statement

The authors have nothing to report.

## Consent

The authors have nothing to report.

## Conflicts of Interest

All authors are employees of ApconiX, an integrated toxicology and ion channel research company.

## Data Availability

The data that support the findings of this study are available on request from the corresponding author. The data are not publicly available due to privacy or ethical restrictions.

## References

[1] D. Cook , D. Brown , R. Alexander , et al., “Lessons Learned From the Fate of AstraZeneca's Drug Pipeline: A Five‐Dimensional Framework,” Nature Reviews. Drug Discovery 13 (2014): 419–431. 24833294 10.1038/nrd4309

[2] S. Authier , J. Arezzo , M. S. Delatte , et al., “Safety Pharmacology Investigations on the Nervous System: An Industry Survey,” Journal of Pharmacological and Toxicological Methods 81 (2016): 37–46. 27263834 10.1016/j.vascn.2016.06.001

[3] A. L. Walker , S. Z. Imam , and R. A. Roberts , “Minireview Drug Discovery and Development: Biomarkers of Neurotoxicity and Neurodegeneration,” (2018), 10.1177/1535370218801309. PMC6434454 30253665

[4] A. Easter , M. E. Bell , J. R. Damewood, Jr. , et al., “Approaches to Seizure Risk Assessment in Preclinical Drug Discovery,” Drug Discovery Today 14 (2009): 876–884. 19545644 10.1016/j.drudis.2009.06.003

[5] P. K. Yip , G. E. Chapman , R. R. Sillito , et al., “Studies on Long Term Behavioural Changes in Group‐Housed Rat Models of Brain and Spinal Cord Injury Using an Automated Home Cage Recording System,” Journal of Neuroscience Methods 321 (2019): 49–63. 30991030 10.1016/j.jneumeth.2019.04.005

[6] A. Easter , T. H. Sharp , J. P. Valentin , and C. E. Pollard , “Pharmacological Validation of a Semi‐Automated In Vitro Hippocampal Brain Slice Assay for Assessment of Seizure Liability,” Journal of Pharmacological and Toxicological Methods 56 (2007): 223–233. 17600733 10.1016/j.vascn.2007.04.008

[7] M. J. Winter , W. S. Redfern , A. J. Hayfield , S. F. Owen , J. P. Valentin , and T. H. Hutchinson , “Validation of a Larval Zebrafish Locomotor Assay for Assessing the Seizure Liability of Early‐Stage Development Drugs,” Journal of Pharmacological and Toxicological Methods 57 (2008): 176–187. 18337127 10.1016/j.vascn.2008.01.004

[8] K. Rockley , R. Roberts , H. Jennings , et al., “An Integrated Approach for Early In Vitro Seizure Prediction Utilizing hiPSC Neurons and Human Ion Channel Assays,” Toxicological Sciences 196 (2023): 126–140. 37632788 10.1093/toxsci/kfad087

[9] W. S. Redfern , L. Carlsson , A. S. Davis , et al., “Relationships Between Preclinical Cardiac Electrophysiology, Clinical QT Interval Prolongation and Torsade de Pointes for a Broad Range of Drugs: Evidence for a Provisional Safety Margin in Drug Development,” Cardiovascular Research 58 (2003): 32–45. 12667944 10.1016/s0008-6363(02)00846-5

[10] D. J. Leishman , M. M. Abernathy , and E. B. Wang , “Revisiting the hERG Safety Margin After 20 Years of Routine hERG Screening,” Journal of Pharmacological and Toxicological Methods 105 (2020): 106900. 32768644 10.1016/j.vascn.2020.106900

[11] E. Kumlien and P. O. Lundberg , “Seizure Risk Associated With Neuroactive Drugs: Data From the WHO Adverse Drug Reactions Database,” Seizure 19 (2010): 69–73. 20036167 10.1016/j.seizure.2009.11.005

[12] A. M. Avila , I. Bebenek , D. L. Mendrick , J. Peretz , J. Yao , and P. C. Brown , “Gaps and Challenges in Nonclinical Assessments of Pharmaceuticals: An FDA/CDER Perspective on Considerations for Development of New Approach Methodologies,” Regulatory Toxicology and Pharmacology 139 (2023): 105345. 36746323 10.1016/j.yrtph.2023.105345

[13] L. Zolkiewski , K. L. Rockley , R. A. Roberts , et al., “Building Structure‐Activity Relationships (SAR) to Avoid Toxicity Due to Unwanted CNS Ion Channel Activity,” (2026). 10.1093/toxsci/kfag094 PMC13493187 42530889

[14] J. P. Valentin , S. Beken , Y. Kanda , et al., “Revisiting ICH S7A after 25 years: Outcomes of a multi‐stakeholder workshop and strategic pillars for modernizing safety pharmacology,” Regulatory Toxicology and Pharmacology 169 (2026): 106096. 41966440 10.1016/j.yrtph.2026.106096

